# Clinical features and surgical management of tuberculous arthritis of the sacroiliac joint: a retrospective analysis of 33 patients

**DOI:** 10.1186/s12893-022-01759-w

**Published:** 2022-08-22

**Authors:** Qi Tian, Linhao Na, Shicong Cao, Zheng Tian, Zheng Guo

**Affiliations:** 1 Xi’an Ninth Hospital, Xi’an, China; 2 Xinjiang Medical University Affiliated First Hospital, Ürümqi, China

**Keywords:** Sacroiliac joint tuberculosis, Posterior and anterior approach, Debridement and curettage

## Abstract

**Background:**

We reviewed 3 different types of tuberculous sacroiliitis via anterior and posterior approaches to determine the efficacy and safety of this surgical approach by describing clinical presentation, imaging, and surgical treatment.

**Methods:**

We reviewed 33 patients with 3 different types of severe tuberculous sacroiliitis, of which 16 patients with anterior iliac abscess underwent anterior debridement. 17 patients underwent posterior debridement. Among them, 5 patients with lumbar tuberculosis underwent lesion debridement through fenestration, joint fusion, and interbody fusion and internal fixation. The mean postoperative follow-up was 16.9 months (12–25 months).Erythrocyte sedimentation rate (ESR), visual analogue scale (VAS) and the Oswestry Disability Index (ODI) were used to judge the postoperative condition and functional recovery.

**Results:**

All patients’ hip, back and lower back pain symptoms were significantly relieved after surgical treatment. At 3 months after operation, the VAS and ODI scores of all patients decreased significantly.

**Conclusion:**

Surgical treatment of tuberculous sacroiliitis should be performed as soon as possible under the adjuvant chemotherapy of anti-tuberculosis drugs. According to the different characteristics of sacroiliac joint tuberculosis, appropriate surgical operations should be adopted according to our classification criteria.

## Introduction

Tuberculosis is the number one cause of death from a single infectious agent according to the World Health Organization’s 2021 Global Tuberculosis Report [1]. The incidence of sacroiliac joint tuberculosis is relatively low, accounting for only 10% of bone and joint tuberculosis. The sacroiliac joints are true synovial joints and are just as susceptible to infection as any other joint [2]. It is mainly manifested as lumbosacral pain and limited mobility of the lower extremities. And only some patients have typical signs of tuberculosis such as low fever and night sweats.

It is difficult to diagnose and differentiate from other diseases of lumbosacral pain [3]. Diagnosis often requires biopsy or bacterial culture based on fine needle aspiration or surgical resection of the lesion. Kim has classified sacroiliac tuberculosis into 4 types [4].Types I and II SJT should be treated with anti-tuberculosis drugs and chemotherapy. For types III and IV, intensive treatment with regular antituberculosis drugs should be combined with debridement and bone graft fusion.

Early diagnosis and stable reconstruction after complete removal of the lesion are the most important treatment methods to prevent the instability of the sacroiliac joint and pelvic ring caused by the lesion [5]. However, there are few research reports on sacroiliac joint tuberculous arthritis, and there is no unified standard for the best treatment. Surgery is mainly divided into open surgery and minimally invasive surgery. And open surgery includes anterior approach; upper anterior portion and posterior approach. Upper anterior portion avoids the iliac muscle from being dissected from the ilium. However, there may be a risk that the lesions cannot be completely removed [6]. Posterior approach avoids the separation of important pelvic neurovascular. However, some scholars believe that anterior approach can reveal the lesion under direct vision and have a larger space to operate the operation. Then the optimal surgical approach for sacroiliac joint tuberculous arthritis remains unclear. The purpose of this study is to classify SJT and adopt corresponding surgical methods to determine its effectiveness.

## Materials and methods

### Design of study

After receiving the written informed consent from participants and approval from hospital’s Ethics Committee, We reviewed 33 cases (15 males and 18 females, classified as type III and IV according to Kim’s classification) of sacroiliac joint tuberculous arthritis (SJT) who were treated in the First Affiliated Hospital of Xinjiang Medical University and underwent anterior debridement from March 2011 to June 2021.

### Settings of the study

The severity of the lesions was assessed by us according to the destruction of the sacroiliac joint surface, the presence of lumbar tuberculosis and the location of the abscess, etc. We divided these patients into 3 categories: A, B and C. Type A is severe sacroiliac joint destruction with or without iliac fossa abscess. Type B is severe sacroiliac joint destruction with posterior iliac abscess, iliac fossa abscess, or no iliac fossa abscess. Type C is tuberculous sacroiliitis with lumbar tuberculosis or paravertebral abscess. Most cases are solitary sacroiliac joint tuberculous arthritis. Only some patients have pulmonary tuberculosis, urinary tuberculosis, pubic tuberculosis and lumbar tuberculosis. We track their treatment outcomes to determine the safety and feasibility of this classification and surgical procedures.

### Study methods

#### Preoperative preparation

After admission, we make a preliminary diagnosis of sacroiliac joint tuberculous arthritis based on typical clinical symptoms combined with blood routine, erythrocyte sedimentation rate, PPD test, test, T-SPOT.TB-DOT test and chemical tests such as anti-tuberculosis antibody. At the same time, we did further verification by combining pelvic frontal X-rays, sacroiliac joint CT and MRI scans. Final diagnosis is confirmed by fine needle aspiration biopsy or intraoperative curettage for culture of Mycobacterium tuberculosis. For patients with high suspicion of sacroiliac joint tuberculosis infection or confirmed by fine needle aspiration biopsy of sacroiliac joint tuberculosis infection. They need immediate quadruple antituberculosis drug treatment. That is, oral isoniazid once a day, a total of 300 mg/d, rifampicin once a day, a total of 450–600 mg/d, pyrazinamide 1–3 times a day, a total of 1500–1750 mg/d, ethambutol Alcohol 1–2 times/d, total 750–1000 mg/d. When symptoms such as body temperature and erythrocyte sedimentation rate are controlled, active nutritional support treatment is required. Anti-tuberculosis drugs were used for about 2 weeks before surgery. Surgery to remove the lesion when the erythrocyte sedimentation rate is below 40 mm/h. Even if the erythrocyte sedimentation rate is still higher than 40 mm/h, SJT with abscess or other vertebral tuberculosis can be treated with surgery after excluding active pulmonary tuberculosis.

### Surgical procedure

#### Anterior approach surgery

**Fig. 1:**
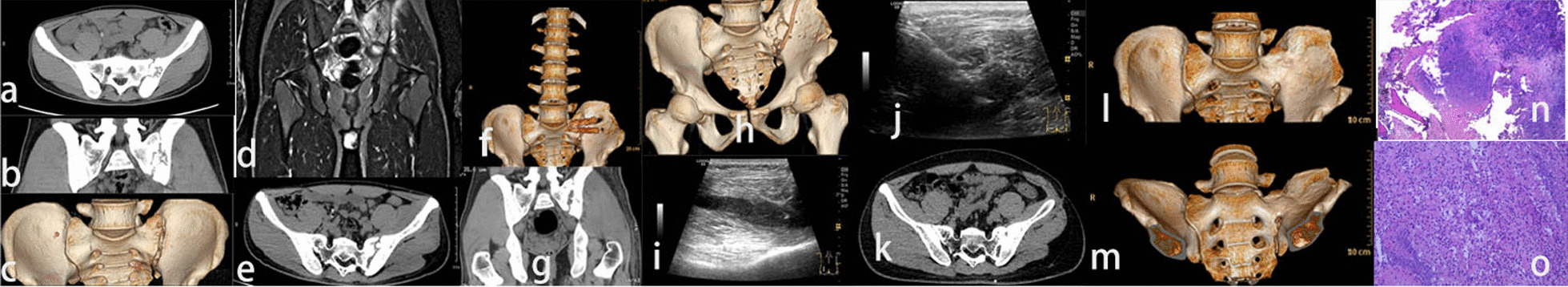
Preoperative CT and MRI, CT at 3 months after operation, CT at 6 months after operation, local B-ultrasound and CT at 12 months after operation of NO.20. **a–d** Bone marrow edema under the left sacroiliac joint, bone destruction under the sacroiliac joint, narrow joint space, irregular and slightly longer T2 signal of the soft tissue below the sacroiliac joint. **e, f** Left sacroiliac joint bone graft stability. **g, h** Left sacroiliac joint The fusion of the iliac joint was further strengthened, and the left gluteus maximus, gluteus medius, and left piriformis were swollen, and there were sheet-like low-density changes inside. **i, j** The visible range of the left buttocks muscle gap was found to be 8.0 in the B-ultrasound 6 months after the operation. 2.3 cm hypoechoic area, 2.4 cm from the body surface. **k–m** CT and 3D reconstruction at 12 months after surgery showed obvious fusion of sacroiliac joints. **n, o** Pathological biopsy showed tuberculous chronic granulomatous inflammation of the left sacroiliac joint with caseation necrosis

For patients with type A SJT with predominant anterior sacroiliac joint destruction with or without anterior abscess or sinus tract, we usually operate through the anterior approach (Fig. 1).

#### Posterior approach surgery

**Fig. 2:**
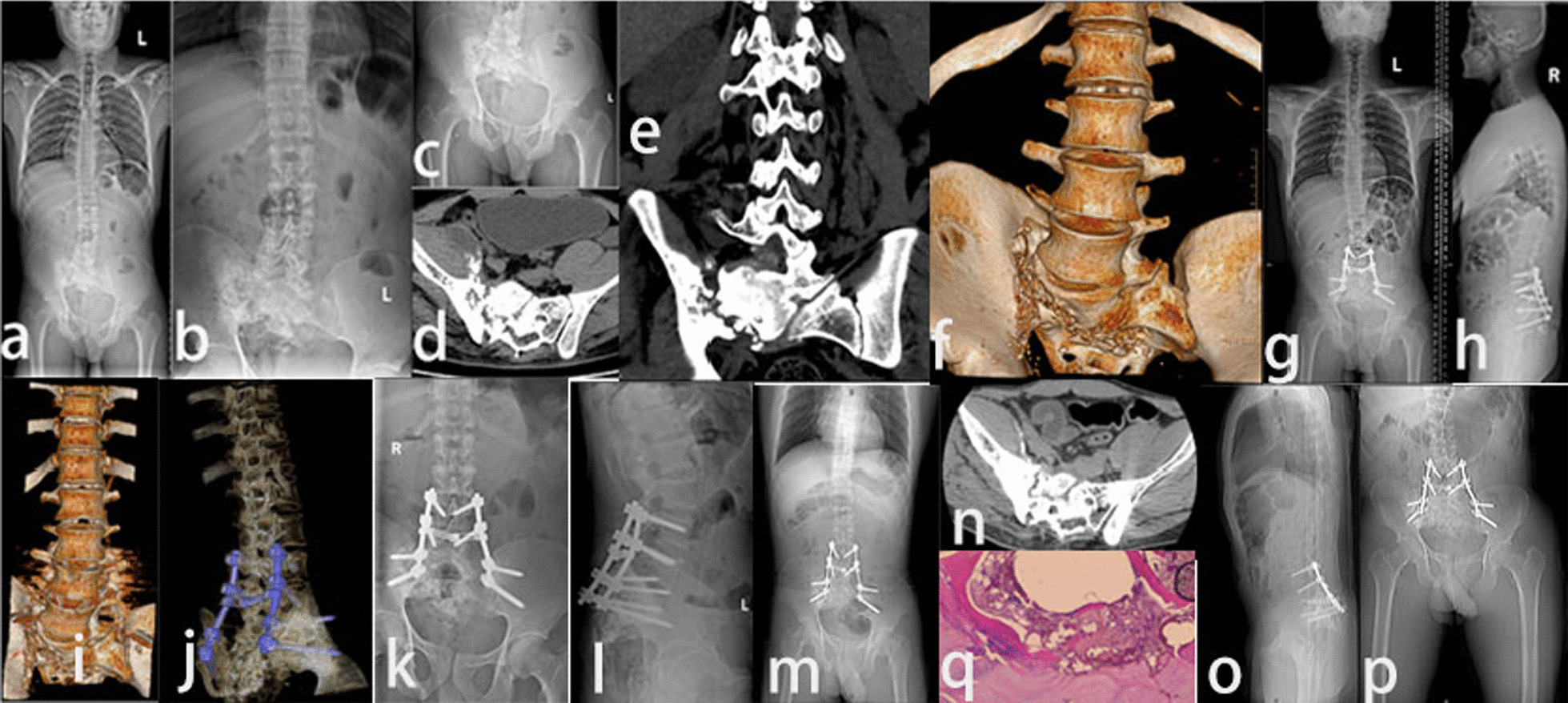
X-rays and CT before and after surgery of NO.5. **a–c** Preoperative X-rays **d–f** Preoperative CT showed the joint space disappeared. Strip calcification can be seen in the spinal canal behind the lumbar 4–5 vertebral body, and the spinal canal is narrowed behind the corresponding intervertebral space. The bone density of the right sacroiliac joint and sacrum is uneven, and multiple worm-like bone destructions can be seen, and multiple spot-like high-density shadows can be seen inside. Similar oval, slightly hypodense shadows in the right psoas muscle, multiple punctate calcifications can be seen on the edge of the lesion and in its interior. The right iliacus muscle was swollen, and punctate high density was seen in the musculature around the pelvic floor. **g, h** Postoperative X-ray. **i**,** j** postoperative CT. **k, l** X-rays 6 months after surgery. **m, n** CT 1 year after operation: the sacroiliac joint is fused, and the joint space disappears. **o**, **p** CT and X-ray films 2 years after surgery showed irregular shape of the right sacroiliac joint, fusion of the sacroiliac joint and disappearance of the joint space. **q** Examination results of surgically removed pathological tissue

For patients with type B SJT with predominant posterior sacroiliac joint destruction with or without a posterior abscess or sinus tract, and type C with lumbosacral tuberculosis, we usually operate through the posterior approach (Fig. 2).

### Postoperative treatment

The drainage tube should be pulled out when the drainage volume is less than 20 ml. Standardized antituberculosis therapy needs to be continued after surgery. The total course of treatment is 18–24 months. Patients need regular review of liver and kidney function to monitor the presence or absence of toxic effects caused by the side effects of anti-tuberculosis drugs during the postoperative period of medication. X-rays should be reviewed regularly after surgery to determine the degree of joint fusion. Computed tomography (CT) and magnetic resonance imaging (MRI) are also reviewed to further confirm the fusion of the sacroiliac joint bone graft if necessary.

### Statistics analysis

Data analysis was performed using SPSS 22.0 (IBM, Chicago, IL, USA). In statistical descriptions, the mean ± standard deviation is used for continuous variables that fit a normal distribution; if they do not fit a normal distribution, the median (interquartile range) is used. The Kolmogorov–Smirnov test was used to test for normality, and the Levene test was used to test the homogeneity of variances. For quantitative data, use ANOVA if the sample is normally distributed and the variance is uniform. If the above conditions are not met, the rank sum test (Kruskal–Wallis test) is used. For qualitative data, the chi-square test or exact probability method was used for the comparison of two groups of binary data and the comparison of two groups of unordered multiclass data, while the rank sum test (Kruskal–Wallis test) was used for the comparison of two groups of ordered multiclass data. The Wilcoxon rank sum test and the Mann–Whitney test were used for VAS and ODI data. A significance level of 0.05 was used.

## Results

Among the 33 patients, there were 6 cases of type III and 27 cases of type IV according to Kim’s classification method. The 16 patients with type A in our study underwent anterior debridement according to the above classification, Lesion debridement of 11 patients with type B via posterior iliac fenestration, Five cases of type C patients with lumbar spine tuberculosis underwent posterior fenestration debridement, arthrodesis and interbody fusion and internal fixation, One patient with type C with anterior paravertebral abscess underwent posterior fenestration debridement and bone graft fusion. Removal of anterior abscess and interbody fusion after secondary surgery (Table 1). Of the patients who underwent type A surgery through the anterior approach, 5 patients were used to fix the surgical area with plates and screws. Plates and screws were not used in patients undergoing type B surgery through the posterior approach. Posterior surgery can achieve solid fixation due to bone grafting through the gap of the iliac fenestration, additional plates and screws are often not required. Anterior approach creates a large bone defect after debridement anterior to the sacroiliac joint, plate screws are needed to promote joint fusion and stabilize the sacroiliac joint.

**Table 1:** Basic information of patients

Case	Age	Sex	Main presentation	Other infections	Side	Course	Type	Approach	Kim’s
1	35	M	Local pain	L4	L	Chronic	B	Posterior	IV
2	32	M	Back pain	L3; L4	L	Chronic	B	Posterior	IV
3	64	M	Local swelling and pain	None	R	Chronic	B	Posterior	IV
4	52	M	Local pain	None	L	Chronic	B	Posterior	IV
5	24	M	Local pain	L5;Left hip joint	R	Chronic	C	Posterior	IV
6	72	F	Local pain	None	L	Chronic	B	Posterior	III
7	38	F	Back pain	None	L	Chronic	B	Posterior	III
8	47	M	Lower limb weakness	L3;L4	R	Chronic	C	Posterior	IV
9	52	F	Local pain	None	R	Acute	B	Posterior	III
10	24	M	Local pain	L1; L2	R	Chronic	C	Posterior	IV
11	31	M	Local pain	None	R	Acute	B	Posterior	IV
12	15	F	Local pain	None	R	Chronic	B	Posterior	IV
13	31	F	Local pain	L5	L	Chronic	C	Posterior	IV
14	33	F	Local pain	None	R	Acute	B	Posterior	III
15	26	F	Local pain	None	R	Chronic	B	Posterior	IV
16	48	F	Local pain	None	L	Chronic	B	Posterior	IV
17	34	F	Back pain	L4;L5,T10;T11, lung	R	Chronic	C	Posterior	IV
18	28	F	lower limb weakness	None	L	Chronic	A	Anterior	III
19	25	M	Local pain	None	R	Acute	A	Anterior	IV
20	25	M	Back pain	None	L	Chronic	A	Anterior	IV
21	17	F	Local pain	None	L	Chronic	A	Anterior	IV
22	21	F	lower limb weakness	None	R	Chronic	A	Anterior	IV
23	20	F	Local pain	None	L	Chronic	A	Anterior	IV
24	16	F	Local pain	None	L	Chronic	A	Anterior	IV
25	19	M	Sciatic pain	None	L	Chronic	A	Anterior	IV
26	15	M	Back pain	None	R	Acute	A	Anterior	IV
27	32	F	Local pain	Pubic bone	L	Chronic	A	Anterior	IV
28	13	F	Local pain	None	R	Chronic	A	Anterior	IV
29	26	F	Back pain	None	R	Acute	A	Anterior	IV
30	23	M	Local pain	None	R	Acute	A	Anterior	IV
31	16	M	Local pain	None	L	Acute	A	Anterior	III
32	36	F	Back pain	None	R	Acute	A	Anterior	IV
33	34	M	Local pain	None	R	Chronic	A	Anterior	IV

One case of severe urinary tract tuberculosis, tuberculous meningitis and severe pulmonary infection occurred 3 months after the anterior approach was excluded. Patients who eventually died of type I respiratory failure and septic shock. Finally, 33 patients with Kim III-IV tuberculous sacroiliitis were followed up. There were 15 males and 18 females. The average age was (31 ± 13.9) years old. All cases were unilateral, with 15 cases on the left side and 18 cases on the right side. The average time from first symptom to diagnosis was 22.7 weeks, the shortest was 2 weeks, and the longest was 27 months. Fifteen of these patients had difficulty walking because they were unable to walk with full weight bearing. Among them, 16 patients had obvious symptoms of tuberculosis such as low-grade fever and night sweats in the afternoon, while the other 17 patients showed no obvious signs of tuberculosis; one patient had nephrectomy due to renal tuberculosis before; 1 patient had pulmonary tuberculosis at the same time; seven patients had lumbar tuberculosis at the same time; 3 patients had palpable masses that could fluctuate on the body surface. And one of them was treated in a local hospital and the pus was removed by puncture. There were two patients with local sinus discharge. There were 13 patients who had received irregular treatments such as acupuncture, massage, anti-inflammatory painkillers and even injection of antibiotics before surgery. Physical examination revealed sacroiliac joint tenderness and pelvic squeezing pain in all patients. Patrick's test was positive. In a state of excessive flexion and extension, passive motion of the affected joint is limited and painful.

The average operation time was 110.3 min (60–250 min). The average anterior approach for type B and C patients was 120.3 min. The average time of posterior approach for patients with type A was 181.3 min; the average blood loss during surgery was 243 ml (50 ~ 1000) ml, The mean blood loss of posterior and anterior procedures was 301.2 ml and 99.7 ml respectively (Only the operation time and blood loss of the sacroiliac joint are compared here). There was no significant difference between the operation time and intraoperative blood loss. The comparison results are (P = 0.213 and P = 0.173). One patient had complications of anterior sinus formation and recurrence 3 months after surgery. Sinus tract was resected and lesions were removed in secondary surgery. Other than that, no other complications were found. One patient suffered a lot of bleeding due to the injury of the artery when the abscess was removed during the operation, and the trouble was finally solved by interventional embolization. The mean erythrocyte sedimentation rate before surgery was 53.7 mm/h (13 ~ 80 mm/h). The blood sedimentation on the 7th postoperative day was 38.7 mm/h (21 ~ 64 mm/h), it basically returned to normal at 3 months after surgery, 11.4 mm/h (4 ~ 20 mm/h) (Table2).

**Table 2:** Outcome of 33 patients received surgical management

Index		Outcome of surgical management (n = 33)		
ESR	Pre-op	Post 7 days	Post 3 month	
	53.7 ± 15.4	38.7 ± 10.7	6.9 ± 1.2	
VAS	Pre-op	Post-op 3 month	6 month	12 months
	6.9 ± 1.2	2.9 ± 0.7	1.8 ± 0.7	1.2 ± 0.4
	P	< 0.001	< 0.001	< 0.001
ODI	56.2 ± 4.7	40.4 ± 4.8	28.8 ± 5.5	4.2 ± 1.5
	P	< 0.001	< 0.001	< 0.001

The normal reference value of ESR in our hospital: < 15 mm/h (male), < 20 mm/h (female)

The sacroiliac joints of all patients were found to have different degrees of damage during the operation. Patient 29 had anterior rupture of the sacroiliac joint capsule with an abscess lesion at the iliopsoas muscle. Patient 20 has an abscess in the groin that develops into a sinus and discharge of pus. Patient No. 5 was accompanied by erosion and destruction of the fifth lumbar vertebra and abscess of the psoas major. His spinal canal was compressed by pus causing lower extremity pain. No serious complications such as joint dislocation were found in all patients during postoperative follow-up.

### Improvement of clinical symptoms

In terms of clinical symptoms, the focus of this study is the patients’ local pain and daily work and life conditions. VAS and ODI scores were used to evaluate the improvement of patients’ clinical symptoms. All patients’ pain symptoms were effectively relieved after surgery. VAS and ODI scores decreased significantly at 3 months postoperatively (Table 2). The VAS and ODI of 33 patients at 3, 6 and 12 months after operation were significantly lower than those before operation. The difference was statistically significant (P < 0.001).The patient had no significant pain or only mild discomfort with activities at 12 months postoperatively. The function of the lower limbs has basically recovered and can meet the needs of daily work and life. All patients were able to walk with full weight bearing, ascend and descend stairs, and perform light exercise at the last follow-up visit. Compared with type A, the preoperative and postoperative ODI patients with lumbar tuberculosis and posterior abscess (types B and C) had no statistical significance (P > 0.05). Except for the VAS score at 6 months after operation, there was no significant difference between the two groups at other time points (P < 0.05). (P = 0.257 before operation. P = 0.075 at 3 months after operation, P = 0.34 at 12 months after operation). Only 1 patient with type A who underwent anterior approach had recurrence 3 months after surgery until the last follow-up (Table 3).

**Table 3:** Outcomes of anterior vs posterior surgery

Outcome		Surgical management and joint fusion (n = 33)				P
Estimate blood loss (ml)	Anterior	181.3 ± 179.3				
	Posterior	301.2 ± 284.6				0.173
Duration of Surgery(min)	Anterior	99.7 ± 33.1				
	Posterior	120.3 ± 54.1				0.213
VAS		Pre-op	3 months	6 months	12 months	
	Anterior	6.7 ± 1.2	3.2 ± 0.6	2.1 ± 0.6	1.3 ± 0.4	
	Posterior	7.2 ± 1.2	2.8 ± 0.6	1.5 ± 0.7	1.1 ± 0.3	
	P^*^	0.257	0.075	0.014	0.34	
ODI	Anterior	56.1 ± 4.2	39.0 ± 4.8	27.6 ± 5.6	4.6 ± 1.6	
	Posterior	56.4 ± 5.1	41.7 ± 4.4	29.9 ± 5.2	4.2 ± 1.5	
	P^*^	0.893	0.117	0.254	0.979	

P* are all results compared to preoperative

## Discussion

The sacroiliac joint consists of anterior and lower synovial structures and posterior 1/3 to 2/3 ligamentous structures; Tuberculosis bacteria penetrate into the cancellous bone and joint synovium with less muscle attachment and abundant blood vessels through the blood circulatory system, causing sacroiliac joint tuberculosis. Partial SJT secondary to adjacent bone and joint tuberculosis commonly seen in lumbar spine tuberculosis. When the bacteria and pus in the synovium further destroy the articular cartilage and the bony structure of the articular surface, it develops into total joint involvement. The lesions are deep and insidious, and the early symptoms and imaging findings are not typical. It is often misdiagnosed as sciatica, discitis, chronic pain syndrome, lumbar disc herniation or spondyloarthritis in the early stage of the disease. The average time from symptom onset to diagnosis in the literature is 14 months [7]. The mean time from first symptom onset to presentation in our study was 22.7 weeks. Localized pain in the groin, buttocks, and back of the thighs is generally the most common clinical presentation. [8–10]. And local symptoms are more severe than systemic symptoms [11]. There were 21 patients with local pain as the main symptom in our study, but only 16 patients (48%) showed typical signs such as afternoon fever and night sweats. Faber’s test and direct tenderness are the most reliable physical findings for evaluating tuberculous sacroiliitis. High false-positive results due to possible compression of the lumbosacral plexus by the abscess during active straight leg raising [2]. Therefore, it is often misdiagnosed as lumbosacral radiculopathy due to the occurrence of lower extremity pain [12]. Sitting, walking, and exercising can make the pain worse as the condition progresses. The joint space is narrow so that too much pus accumulate. When the pus breaks through the joint capsule and overflows, the joint pain is partially relieved due to the reduced pressure and it spreads along the weak tissue space to form a sinus tract. Tuberculous sacroiliitis can be divided into caseous necrosis type and proliferative type in pathology. The former often involves the surrounding soft tissue and causes caseous necrosis and sequestrum formation. The necrotic material liquefies to form abscesses and sinus tracts. The proliferative type is relatively rare, mainly forming granulation tuberculosis tissue and destroying the trabeculae under the bone [13]. X-ray and CT are mainly used for imaging examination. Since the sacroiliac joint has an included angle of 15° with the sagittal plane of the longitudinal axis of the body, the X-ray should take a plain film of the pelvis and the sacroiliac joint with the affected side elevated by 15° in the supine position [14]. CT and MRI are more helpful in the early diagnosis of tuberculous sacroiliitis. The advantage of CT is to show fine synovial thickening, articular surface destruction, small bone abscesses, sequestrum, cystic degeneration, and osteosclerosis. [7, 15]. MRI can help show the location and size of the abscess. [16]. But the final diagnosis is by fine needle aspiration and intraoperative biopsy.

The purpose of surgery for tuberculous sacroiliitis is to remove the tuberculosis and fuse the sacroiliac joints [17]. Conventional surgical methods are divided into anterior approach and posterior approach. We divide it into 3 surgical methods according to the characteristics of the patient’s lesions. Anterior approach is chosen when SJT is type A with iliac fossa abscess. Anterior surgery helps to preserve the stability of the joint because the lesions of joint destruction are generally in the anterior half, and the posterior ligaments and other tissues are not violated. Anterior approach surgery is usually done in the posterior peritoneum for debridement. The sacroiliac joint can be clearly exposed under direct vision after incision of the anterior ligament and joint capsule. There is enough space in the front to operate, which facilitates the insertion of internal fixation. However, due to the existence of large blood vessels and lumbosacral plexus inside the pelvis, the operation may be difficult and the amount of bleeding may be large. For category B and C SJT with hip abscess we choose posterior approach. Posterior approach is safe and easy to operate. However, incision of the posterior ligament and joint capsule of the sacroiliac joint will destroy the stability of the ligament around the joint, and now it is often used to open the window through the iliac bone and perform curettage of the lesion. However, the disadvantage of this operation is that the lesion cannot be cured under complete direct vision, and the operating space is small. Generally, the stability of the bone fragment can be maintained by suturing the tissue after bone grafting. If the bone fragment cannot be stabilized, an internal fixation device should be used to restore the stability of the joint. Patients without internal fixation with steel plates should be bundled with compression such as braces or bandages for 3 weeks after surgery to facilitate the fusion of implanted bone fragments. For C-type SJT with lumbar tuberculosis, posterior surgical removal of sacroiliac lesions and debridement of lumbar tuberculosis and one-stage pedicle screw therapy can achieve stable fusion of the intervertebral and sacroiliac joints at the same time. The base of the posterior superior iliac spine is the best position for iliac screw fixation of the iliac bone. The iliac bone screw should be inserted in the cylindrical area above the greater sciatic notch along the direction of the acetabulum. This is not only safe and easy, but also can obtain maximum holding force [5].

All patients’ symptoms and activity limitation were effectively relieved after surgery. Their VAS and ODI at 3, 6, and 12 months postoperatively were all meaningful compared to preoperatively. Erythrocyte sedimentation rate decreased to normal levels at 3 months postoperatively. There was no significant difference in intraoperative blood loss and operation time between anterior and posterior surgery. Quiescent sacroiliac joint tuberculosis requires posterior surgery to avoid greater trauma, while for SJT with sacroiliac abscesses, anterior abscess removal is required. The study by Zhu et al. showed that posterior fenestration for SJT is a safe and feasible modality [18]. There was no significant difference in VAS and ODI between anterior and posterior procedures at 3, 6, and 12 months postoperatively, so both procedures are effective treatments for SJT. Because there is an important neurovascular network in front of the sacroiliac joint, and the lesions are deep, it is prone to iatrogenic trauma. The iliac bone behind the sacroiliac joint is thick, superficial, and has no important nerves and blood vessels, so the risk of exposure is small. Sinus of the groin usually occurs after incision and drainage of an anterior abscess [19]. Many scholars suggest that sacroiliitis should be removed by a posterior approach for bone grafting [20]. Our study also found that the anterior approach may have more complications. A patient who underwent anterior approach surgery inadvertently injured the artery during the removal of the abscess, and finally stopped the bleeding by interventional embolization. Another patient with anterior approach developed a sinus tract at 3 months after surgery. No complications were found in patients with the posterior approach. However, performing surgery according to our SJT classification can ensure complete removal of the lesion. After all, complete removal of the lesion is the ultimate goal of surgery.

The following points need to be paid attention to during the operation: 1 Choose the appropriate surgical approach according to the location and characteristics of the lesion before the operation. Thorough debridement is the most powerful guarantee to prevent surgical failure. Incomplete debridement can easily lead to recurrence of infection. When removing lesions and abscesses during the operation, careful and careful debridement should be combined with imaging studies. Soft silicone tubes should be fully used for irrigation and drainage, and if necessary, they should be removed. Part of the lamina on the affected side to remove the presacral abscess from the back to the front, and avoid the use of sharp instruments to damage the presacral blood vessels and the lumbosacral plexus. 2 For anterior approach surgery, pay attention to flexing the hip and knee joint to relax the L4 and L5 nerve roots. 3 In the anterior approach, the iliac muscle should be stripped away with other soft tissues of the pelvis and pulled to the inside together with blunt retractors to protect the superior gluteal artery and vein and the lumbosacral trunk. The stripping range on the sacral side should not exceed 20 mm inside the sacroiliac joint [21, 22]. 4 The iliac muscle should be stripped under the periosteum through the anterior approach, and bone wax is used to stop bleeding when the feeding vessels bleed. 5 The gauze should be wrapped with fingers to separate the tissue carefully and gently when the lesions are exposed to the sacroiliac joint during the operation; 6 For those with sinus tract, firstly remove the abscess lesions inward along the sinus tract and search for the bone cavity at the sacroiliac joint, and finally extend to the intra-articular lesions for complete curettage. 7 As it is difficult to completely close out the lesions by fenestration and curettage through the posterior approach, anhydrous ethanol, carbolic acid and hydrogen peroxide should be used together. 8 Due to the large residual cavity after curettage, adequate bone grafting is required to facilitate fusion. We recommend fenestration and autogenous ilium. 9 The combination of anti-tuberculosis drug chemotherapy and surgery must be adhered to, and the principles of sufficient dose, combination, whole process and regularity must be followed.

Generally speaking, SJT patients with large abscess cavity, thin pus and poor constitution have a high recurrence rate after surgery. However, patients with small abscess cavity, thick pus and strong constitution have less chance of recurrence and good postoperative curative effect [23]. Therefore, it should be noted during the operation that the sinus tract and abscess cavity should be eliminated first, and then the joint space lesions should be completely cured. Finally, place a negative pressure drainage tube in the operation area and continue to adhere to the drug consolidation therapy for 1–1.5 years after surgery. Tuberculosis recurrence should be prevented in patients with pulmonary tuberculosis or massive bone destruction [7]. In our study, there was 1 patient with anterior metamorphic surgery who had recurrence 3 months after surgery, which was considered to be caused by the formation of sinus tract after surgery. Therefore, it is necessary not only to select the appropriate surgery according to our disease classification, but also to carefully remove the soft tissue lesions that may be infected; all patients were treated with standard quadruple antituberculosis drugs before and after surgery, and the total course of treatment was 18–24 months.

The advantage of this study is that the corresponding surgery is selected according to the different characteristics of Kim’s type III-IV SJT abscesses and lesions, and similar studies are relatively few. This provides some experience in the treatment of sacroiliac joint tuberculous arthritis.

This study has certain limitations. This is a retrospective study, and the number of cases is not large enough. Therefore, some prospective studies and studies with larger sample sizes are needed in future studies. In addition, although anterior surgery is somewhat cumbersome because of the important blood vessels and nerves in the anterior. However, anterior surgery is the most promising way to completely remove the lesions for type A SJT. Therefore, each SJT patient needs to synthesize its unique lesion characteristics and choose the appropriate surgical method.

## Conclusion

Due to the insidious onset of tuberculous sacroiliitis, it is often misdiagnosed in the early stage and receives informal treatment. Therefore, tuberculous sacroiliitis cannot be easily ignored when the patient presents with pain in the groin, buttocks, and around the sacroiliac and an abnormally high erythrocyte sedimentation rate. A detailed history and careful examination of the sacroiliac joints are key to the diagnosis. It is highly suggestive of tuberculosis when X-ray and CT scans show sacroiliac joint calcification, joint space enlargement, and articular surface destruction. The diagnosis should be confirmed by fine needle aspiration biopsy in the early stages of infection. Chemotherapy with standard anti-TB drugs should be aggressively administered when TB infection is identified.Surgery should be performed as early as possible to facilitate fusion of the sacroiliac joints after control of tuberculosis infection. Posterior fenestration with minimal secondary damage to the joint is the first choice for stationary SJT without abscess. Anterior approach is required for SJT with an abscess in the anterior sacroiliac.

## Data Availability

The data sets generated and analyzed during the current study are not publicly available due to restrictions on ethical approvals involving patient data and anonymity but can be obtained from the corresponding authors as reasonably required.
